# Multicenter comparison of PEG-IFN α2a or α2b plus ribavirin for treatment-naïve HCV patient in Korean population

**DOI:** 10.1186/1471-230X-13-74

**Published:** 2013-04-29

**Authors:** Young-Joo Jin, Jin-Woo Lee, Jung il Lee, Sang Hoon Park, Choong Kee Park, Young Seok Kim, Sook-Hyang Jeong, Yun Soo Kim, Ju Hyun Kim, Seong Gyu Hwang, Kyu Sung Rim, Hyung Joon Yim, Jae Youn Cheong, Sung Won Cho, June Sung Lee, Young Min Park, Jeong Won Jang, Chun Kyon Lee, Joo Hyun Sohn, Jin Mo Yang, Seungbong Han

**Affiliations:** 1 Department of Internal Medicine, Incheon, South Korea; 2 Inha University School of Medicine, Incheon, South Korea; 3 Hallym University Kangnam Sacred Heart Hospital, Seoul, South Korea; 4 Hallym University Sacred Heart Hospital, Anyang, South Korea; 5 Soonchunhyang University College of Medicine, Bucheon, Korea; 6 Seoul National University Bundang Hospital, Seongnam, South Korea; 7 Gachon University Gil Medical Center, Incheon, South Korea; 8 CHA Bundang Medical Center, CHA University, Seongnam, South Korea; 9 Korea University College of Medicine, Ansan, South Korea; 10 Ajou University School of Medicine, Suwon, South Korea; 11 Ilsan Paik Hospital, Inje University College of Medicine, Goyang, South Korea; 12 Bundang Jesaeng General Hospital, Daejin Medical Center, Seongnam, South Korea; 13 Incheon St. Mary's Hospital, The Catholic University of Korea College of Medicine, Incheon, South Korea; 14 National Health Insurance Corporation Ilsan Hospital, Goyang, Suwon, Republic of Korea; 15 Guri Hospital, Hanyang University College of Medicine, Guri, St.Vincent Hospital, The Catholic University College of Medicine, Suwon, Republic of Korea; 16 Department of Biostatistics and Clinical Epidemiology, Asan Medical Center, University of Ulsan College of Medicine, Seoul, Republic of Korea; 17 Department of Internal Medicine, Inha University Hospital, Inha University School of Medicine, 27 Inhang-ro, Jung-gu, Incheon, 400-711, Republic of Korea

**Keywords:** Chronic hepatitis C, Pegylated interferon alfa-2a, Pegylated interferon alfa-2b, Ribavirin, Sustained virological response

## Abstract

**Background:**

Two recent Italian studies suggested that Pegylated-interferon (PEG-IFN) alfa-2a achieves a higher sustained virological response (SVR) rate than PEG-IFN alfa-2b. We intended to compare the efficacy and safety of PEG-IFN alfa-2a with those of PEG-IFN alfa-2b in Korean patients with chronic hepatitis C virus (HCV).

**Methods:**

This retrospective, multi-center trial was conducted on 661 treatment-naïve chronic HCV patients. Patients received PEG-IFN alfa-2a (180 μg/week; n=402) or PEG-IFN alfa-2b (1.5 μg/kg/week; n=259) with ribavirin (800–1200 mg/day) for 24 or 48 weeks according to HCV genotypes.

**Results:**

Early virologic response and sustained virologic response (SVR) rates were not significantly different between two PEG-IFN groups both in patients with HCV genotype 1 (all *P*-values>0.05) and 2/3 (all *P*-values>0.05). SVR rates were not different between two groups in each categorized baseline characteristics: age (years) (≤50 and >50), HCV viral load (IU/mL) (≤7×10^5^ and >7×10^5^), and hepatic fibrosis (F0-2 and F3-4) (all *P*-values >0.05). In additional analysis for 480 patients who sufficiently complied with treatment doses and duration (80/80/80 rule) and propensity-score matched analysis, SVR rates were not different between two groups both in patients with HCV genotype 1 and 2/3 (all *P*-values >0.05). Adverse event rates were similar between two groups.

**Conclusions:**

Unlike the Western data, efficacy and safety of PEG-IFN alfa-2a were similar to those of PEG-IFN alfa-2b in chronically HCV-infected Korean patients regardless of age, HCV viral load, and hepatic fibrosis.

## Background

Chronic hepatitis C virus (HCV) infection is a major health problem globally [1,2]. Because chronic HCV infection is a leading cause of liver cirrhosis or hepatocellular carcinoma, and is the principal indication for liver transplantation [2-4], the selection of the most effective and safe drugs may be pivotal for the treatment of chronically HCV-infected patients to reduce HCV-related morbidity or mortality.

Consensus guidelines recommend the use of either Pegylated interferon (PEG-IFN) alfa-2a (40KD) or PEG-IFN alfa-2b (12KD) both plus ribavirin for the treatment of chronic HCV infection [2,5,6]. Previous non-comparative studies have shown that these regimens have similar efficacy and safety [7,8], despite the different pharmacokinetic and pharmacodynamic properties of PEG-IFN alfa-2a and alfa-2b [9,10]. Although recent large-scale randomized trials conducted in the West have showed that these two PEG-IFNs are similar in terms of efficacy and tolerability, these trial were limited to genotype 1 chronic HCV patients or HIV co-infected patients [11,12], In contrast, two comparative Italian studies and a Cochrane meta-analysis for randomized trials suggested that sustained virological response (SVR) rate of PEG-IFN alfa-2a is superior to that of PEG-IFN alfa-2b [13-15].

However, relative efficacy of PEG-IFN alfa-2a plus ribavirin compared with PEG-IFN alfa-2b plus ribavirin in Korean chronic HCV patients remains unclear. Furthermore, it is not known whether the higher SVR rates by PEG-IFN alfa-2a compared with that by PEG-IFN alfa-2b can be achieved in chronic HCV patients who live in Asia region such as Korea, where better outcomes of anti-HCV treatment can be achieved than Caucasian area when patients were treated with the same regimen [16].

In this large-scale multicenter study, therefore, we aimed to compare the therapeutic efficacy and tolerability of PEG-IFN alfa-2a and PEG-IFN alfa-2b in combination with ribavirin in treatment-naive Korean patients with chronic HCV infection.

## Methods

### Study subjects

Between January 2000 and September 2008, 694 consecutive adult patients were diagnosed as having chronic HCV infection at 14 referral hospitals in Korea. The diagnosis of chronic HCV infection was made based on the American Association for the Study of Liver Diseases (AASLD) guideline [5]. All were positive for anti-HCV antibody test with/without an elevation of serum alanine aminotransferase (ALT) levels for more than 6 months. Moreover, baseline quantitative HCV-RNA test were performed in all patients, and they were ≥18 years old. None of these patients had received interferon or PEG-IFN previously for the treatment of chronic HCV infection.

Of these 694 patients, 33 were excluded due to hepatitis B virus (n=27) and human immunodeficiency virus (n=6) co-infection. Therefore, our multicenter cohort consisted of 661 patients and a retrospective database was obtained from these patients. The study protocol was approved by the Institutional Review Boards at the hospitals concerned.

### Recruitment of clinical data

We evaluated following databases before chronic HCV treatment: age, gender, weight, body mass index; international normalized ratio (INR); ALT, total bilirubin, albumin, and creatinine; white blood cell count, absolute neutrophil count, hemoglobin, and platelet count; viral hepatitis test with HBsAg, and anti-HBV core IgM, anti-hepatitis A IgM, and anti-HCV antibodies; serologic tests for HIV; quantitative HCV RNA titer (IU/mL) and HCV genotype. Serum HCV RNA titers were determined using a quantitative PCR assay (Cobas Amplicor HCV Monitor Test v2.0, Roche Diagnostics, Basel, Switzerland) or Abbott real-time kit (Abbott Molecular Inc., Abbott Park, IL, USA), and HCV genotypes were determined by INNO-LiPA (Innogenetics NV, Gent, Belgium) HCV test. If available, histologic specimens of liver tissues were also evaluated. Fibrosis stages in liver specimens were established by pathologists based on previously published guidelines [17], and significant fibrosis was defined as a fibrosis stage of ≥F3 based on METAVIR scoring system.

During antiviral treatment, WBC, ANC, hemoglobin, platelet count, HCV RNA titer, and serum ALT levels were recruited at 4, 12, and 24 weeks, respectively, in genotype 2/3 (2 or 3) HCV patients, and at 4, 12, 24, and 48 weeks, respectively, in genotype non-2/3 HCV patients. Qualitative HCV RNA using Abbott Diagnostic Division (Abbott Park, IL, USA) or Biocore HCV RT-PCR version 2.0 (BioCore, Seoul, Korea) was also assessed at treatment cessation. At 24 weeks after treatment completion, these laboratory data were also obtained for all patients.

### Treatment for chronic HCV infection

A once weekly subcutaneous injection regimen of PEG-IFN α-2a (Pegasys; Roche, Basel, Switz, 180 mcg) or PEG-IFN α-2b (PegIntron; Schering Plough Corp., Kenilworth, N.J., USA, 1.5 mcg/kg) was planned for 24 weeks in patients with HCV genotype 2/3 and for 48 weeks in those with HCV genotype non-2/3 according to the AASLD guidelines [5]. In addition, all patients received daily oral ribavirin (Rebetol; Schering Plough Corp., Kenilworth, N.J., USA) and RBV dosage was determined by body weight according to HCV genotype in both PEG-IFN groups: 800 mg/day for genotype 2/3 and 1,000 mg/day (body weight ≤75 kg) or 1,200 mg/day (>75 kg) for genotype non 2/3 [5].

Doses of PEG-IFN and ribavirin during treatment were modified according to the AASLD guideline [5]. Briefly, the dosage of PEG-IFN was reduced by half if the neutrophil count decreased to ≤750/mm^3^ or the platelet count decreased to ≤50,000/mm^3^. PEG-IFN treatment was discontinued if the neutrophil count rose to ≤500/mm^3^ or the platelet count reduced to ≤25,000/mm^3^. The dosage of ribavirin was reduced by 200 mg/day if the hemoglobin level decreased to ≤10 g/dL, and ribavirin was discontinued if the hemoglobin level decreased to ≤8.5 g/dL.

### Evaluation of efficacy

The therapeutic efficacy of the PEG-IFN regimens were assessed using SVR rates, which was defined as undetectable HCV-RNA at 24 weeks after treatment cessation. Rapid virological response (RVR) and end-of-treatment response (ETR) were defined as HCV RNA negative at treatment week 4 and at the end of treatment, respectively. Early virological response (EVR) was defined as a ≥2 log reduction in HCV RNA level versus baseline (partial EVR) or a HCV RNA negative status at treatment week 12 (complete EVR) [5].

### Evaluation of safety

The following treatment-related adverse events (AEs) were assessed: flu-like symptoms, emotional friability, alopecia, skin reaction, and gastrointestinal disorders. Early drug discontinuation due to an AE was also included in the safety assessment. AEs were graded as I, II, or III according to drug dose modification, that is grade 1 was defined as AE without reducing the drug dose, grade II as AE with drug dose reduction; and grade III as AE leading to early drug discontinuation. Serious AEs, such as treatment-related severe infections, were also assessed. Patients who took at least 80% of the 2 prescribed drugs for at least 80% of the scheduled time (80/80/80 rule) were considered to be adherent [18].

### Statistical analysis

The baseline clinical characteristics of patients are expressed as mean (standard deviation), median (range), or frequencies. Differences between categorical or continuous variables were analyzed using the *chi*-square test, Fisher’s exact test, or the Student’s *t* test. Intention to treat (ITT) analysis was performed to compare the efficacy of the two regimens, and in order to avoid various misleading artifacts. Patients who were lost to follow up after initial treatment were considered as nonresponders. Additional analysis was performed in patients who sufficiently complied with the treatment schedule (the 80/80/80 rule) in terms of anti-HCV treatment to estimate pure treatment effects. Furthermore, propensity-score matching for age, gender, HCV genotype, HCV RNA titer, and serum ALT level between two groups was performed to control potential confounding factors. Safety outcomes are reported for all patients. A two-tailed *P*-value less than 0.05 was considered statistically significant in all analyses. Statistical analyses were performed using SPSS v18.0 (SPSS Inc, Chicago, IL).

## Results

### Baseline demographics and characteristics

Baseline characteristics of the 661 patients are summarized in Table 1. Of the 661 patients, 416 patients were genotype 1, and 235 patients were genotype 2 or 3. Of the 416 patients with genotype 1, 254 (61.1%) patients received PEG-IFN alfa-2a plus ribavirin (the PEG-IFN alfa-2a group) and the remaining 162 (38.9%) patients received PEG-IFN alfa-2b plus ribavirin (the PEG-IFN alfa-2b group). Of the 235 patients with genotype 2/3, 141 (60%) patients were PEG-IFN alfa-2a group and the remaining 94 (40%) patients were PEG-IFN alfa-2b group.

**Table 1:** Characteristics of patients with chronic HCV infection

**Variables**	**PEG-IFN alfa-2a**	**PEG-IFN alfa-2b**	P*
	**(n=402, 60.8%)**	**(n=259, 39.2%)**	
**Genotype 1 (n=416), n (%)**	**254 (61.1)**	**162 (38.9)**	
Age, years^†^	49 ± 11	51 ± 11	0.26
>50 years, n (%)	121 (47.6)	83 (51.2)	0.48
Gender (male), n (%)	154 (60.6)	100 (61.7)	0.74
Weight (Kg)	67 ± 12	66 ± 11	0.93
≥ 75 kg, n (%)	48 (18.9)	34 (21.0)	0.62
BMI (Kg/m^2^) ^†^	24.3 ± 3.2	24.4 ± 3.1	0.81
WBC (/mm^3^) ^†^	5.5 ± 2.1	5.2 ± 1.6	0.11
Hemoglobin (g/dL) ^†^	13.9 ± 1.8	13.9 ± 1.7	0.72
Platelet (x10^3^/mm^3^) ^†^	173 ± 72	167 ± 71	0.46
ALT (IU/L) ^†^	104 ± 86	94 ± 80	0.26
HCV RNA (IU/mL) ^†^	2.9×10^6^ ± 1.1×10^6^	4.8×10^6^ ± 1.9×10^6^	0.24
Fibrosis (stage), F0-2/F3-4, n (%)^§^	62/43 (59.0/41.0)	18/6 (75.0/25.0)	0.17
Adherence, ≥ 80%/<80%	171/83 (67.3/32.7)	115/47(71.0/29.0)	0.45
**Genotype 2 or 3 (n=235), n (%)**	**141 (60.0)**	**94 (40.0)**	
Age, years^†^	49 ± 12	51 ± 11	0.19
>50 years, n (%)	61 (43.3)	49 (52.1)	0.19
Gender (male), n (%)	79 (56.0)	54 (57.4)	0.89
Weight (Kg)	65 ± 11	64 ± 10	0.51
≥ 75 kg, n (%)	25 (17.7)	11 (11.7)	0.27
BMI (Kg/m^2^) ^†^	23.8 ± 3.3	23.9 ± 2.7	0.98
WBC (/mm^3^) ^†^	5.3 ± 1.5	5.1 ± 1.8	0.46
Hemoglobin (g/dL) ^†^	14.2 ± 1.6	13.7 ± 1.5	0.06
Platelet (x10^3^/mm^3^) ^†^	177 ± 62	168 ± 65	0.32
ALT (IU/L) ^†^	98 ± 88	90 ± 89	0.51
HCV RNA (IU/mL) ^†^	2.0×10^6^ ± 1.9×10^6^	1.9×10^6^ ± 1.0×10^6^	0.83
Fibrosis (stage), F0-2/F3-4, n (%)^#^	51/10 (83.6/16.4)	8/5 (61.5/38.5)	0.12
Adherence, ≥ 80%/<80%	107/34 (75.9/24.1)	78/16 (83.0/17.0)	0.26
**Genotype others (n=10), n (%)**	**7(70.0)**	**3 (30.0)**	

Abbreviation: *HCV*, hepatitis C virus; *BMI*, body mass index; *ALT*, alanine aminotransferase; *PEG-IFN*, peginterferon; F0-2, no or insignificant fibrosis; F3-4, significant fibrosis or cirrhosis.

^†^mean ± standard deviation.

^§, # ^Data were available only in 129 (genotype 1) and 74 (genotype 2/3) patients who underwent liver biopsy before initiation of antiviral treatment.

At the time of anti-HCV treatment, most baseline demographic and clinical characteristics including age, gender, BMI, serum ALT levels, and HCV RNA titers were similar between two groups. A liver biopsy was performed in 129 (31%) of 416 patients with genotype 1, and 74 (31.5%) of 235 patients with genotype 2/3. Histologic data about hepatic fibrosis was available for these patients. Histologic distributions of significant hepatic fibrosis (F3 or F4) were similar between two groups both in patients with genotype 1 (*P*=0.17) and genotype 2/3 (*P*=0.12) (Table 1).

### Virologic response

**Figure 1:**
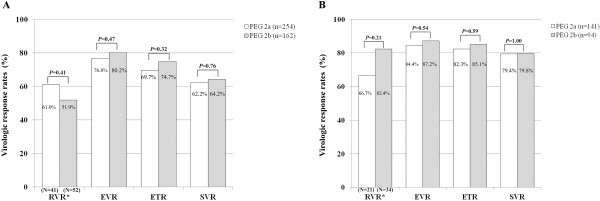
**Intension to treat analysis for patients with genotype 1 and genotype 2/3. **RVR, EVR, ETR, and SVR rates were not statistically different between the two PEG-IFN groups in patients with genotype 1 (**A**) and genotype 2/3 (**B**).

By intention-to-treat analysis, in 416 patients with genotype 1, EVR (76.8% *vs.* 80.2%, *P*=0.47), ETR (69.7% *vs.* 74.7%, *P*=0.32), and SVR (62.2% *vs.* 64.2%, *P*=0.76) were not different between two PEG-IFN groups (Figure 1A). In 235 patients with genotype 2/3, EVR (84.4% *vs.* 87.2%, *P*=0.54), ETR (82.3% *vs.* 85.1%, *P*=0.59), and SVR (79.4% *vs.* 79.8%, *P*=1.00) were not different between two PEG-IFN groups (Figure 1B).

Because the concept of RVR was adopted relatively recently, only 93 (22.4%) and 55 (23.4%) patients had available RVR data in patients with genotype 1 and genotype 2/3, respectively. Of these patients, RVR were not different between two PEG-IFN groups in patients with genotype 1 (61.0% *vs.* 51.9%, *P*=0.41) and genotype 2/3 (66.7% *vs.* 82.4%, *P*=0.21) (Figure 1A, B). In subgroup analysis for SVR in patients who had available RVR data, SVR rates were higher in patients with genotype 1 (43.9% *vs.* 84.6%, *P*<0.01) and genotype 2/3 (46.2% *vs.* 95.2%, *P*<0.01), respectively, who achieved RVR than those who did not. Furthermore, SVR rates were not different between two PEG-IFNs groups even after stratification of the baseline characteristics: age (years) (≤50 and >50), HCV viral load (IU/mL) (≤7×10^5^ and >7×10^5^), or hepatic fibrosis (F0-2 and F3-4) (*P*-values for each >0.05) in patients with HCV genotype 1 and genotype 2/3, respectively (Table 2).

**Table 2:** Comparison of SVR rates between two PEG-IFN groups according to categorized variables

**Genotype 1**	**PEG-IFN alfa-2a**	**PEG-IFN alfa-2b**	P*
**(n=416)**	**(n=254)**	**(n=162)**	
SVR, n (%)	158 (62.2)	104 (64.2)	0.76
Age, n (%)			
≤50 years (n=212)	88/133 (66.2)	61//79 (77.2)	0.12
>50 years (n=204)	70/121 (57.9)	43/83 (51.8)	0.47
HCV RNA (IU/mL), n (%)			
≤7×10^5^(n=149)	76/117 (65.0)	46/67 (68.7)	0.63
>7×10^5^(n=232)	82/137 (59.9)	58/95 (61.1)	0.89
Liver fibrosis, n (%) ^†^			
F 0–2 (n=80)	48/62 (77.4)	11/18 (61.1)	0.22
F 3–4 (n=49)	18/43 (41.9)	4/6 (66.7)	0.39
**Genotype 2/3**	**PEG-IFN alfa-2a**	**PEG-IFN alfa-2b**	**0.29**
**(n=235)**	**(n=141)**	**(n=94)**	
SVR, n (%)	112 (79.4)	75 (79.8)	1.00
Age, n (%)			
≤50 years (n=125)	71/80 (88.8)	38/45 (84.4)	0.58
>50 years(n=110)	41/61 (67.2)	37/49 (75.5)	0.40
HCV RNA (IU/mL), n (%)			
≤7×10^5 ^(n=137)	63/81(77.8)	49/56 (87.5)	0.18
>7×10^5 ^(n=98)	49/60 (81.7)	26/38 (68.4)	0.15
Liver fibrosis, n (%) ^‡^			
F 0–2 (n=59)	42/51 (82.4)	7/8 (87.5)	1.00
F 3–4 (n=15)	7/10 (70.0)	3/5 (60.0)	1.00

Abbreviation: *HCV*, hepatitis C virus; *PEG-IFN*, peginterferon; *SVR*, sustained virologic response.

^†, ‡^ Data were available in 129 and 74 patients, respectively. If the number of patients was smaller than 5, Fisher’s exact test was used.

**Figure 2:**
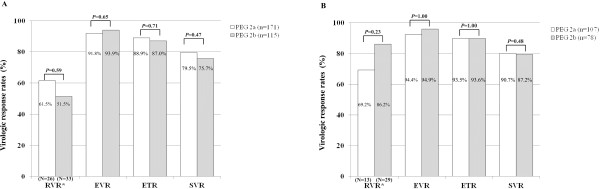
**Analysis in patients who were sufficiently adherent (80/80/80 rule) to treatment. **RVR, EVR, ETR, and SVR rates were not statistically different between the two PEG-IFN groups in patients with genotype 1 (**A**) and genotype 2/3 (**B**).

To compare the pure treatment effects by two PEG-IFNs, 286 patients with genotype 1 and 185 patients with genotype 2/3 who sufficiently complied with the treatment schedule (80/80/80 rule) were additionally analyzed (Table 3). In this analysis, age, gender, BMI, and serum ALT, and HCV RNA titer were similar between two groups (*P*-values for each >0.05). Both in patients with HCV genotype 1 and genotype 2/3, EVR, ETR, and SVR rates did not differ significantly between the two treatment groups (*P*-values for each >0.05) (Figure 2A, B). RVR data were available only for 59 (20.6%) patients with genotype 1 and for 42 (22.7%) patients with genotype 2/3, and RVR rates did not significantly differ between the two treatments groups in those with genotype 1 (*P*=0.59) and in those with genotype 2/3 (*P*=0.23) (Figure 2A, B).

**Table 3:** Characteristics of patients who met 80/80/80 rule and were candidate for propensity score matching

**Patients who met**	**Total**	**PEG-IFN alfa-2a**	**PEG-IFN alfa- 2b**	P*
**80/80/80 rule**				
Genotype 1, n (%)	286	171 (59.8)	115 (40.2)	0.45
Age, years^†^	49 ± 11	48 ± 11	49 ± 11	0.52
BMI (Kg/m^2^) ^†^	24.6 ± 3.1	24.8 ± 3.2	24.3 ± 2.9	0.29
ALT (IU/L) ^†^	100 ± 81	103 ± 80	97 ± 84	0.51
HCV RNA (IU/mL) ^†^	3.7×10^6^±1.6×10^6^	2.4×10^6^±1.5×10^6^	5.6×10^6^±2.2×10^6^	0.15
F3-4 Fibrosis stage, n (%) ^§^	29/94 (30.9)	26/75 (34.7)	3/19 (15.8)	0.17
Genotype 2/3, n (%)	185	107 (57.8)	78 (42.2)	
Age, years^†^	49 ± 12	48 ± 12	50 ± 11	0.16
BMI (Kg/m^2^) ^†^	23.9 ± 2.7	24.3 ± 3.1	23.6 ± 2.4	0.16
ALT (IU/L) ^†^	97 ± 94	101 ± 88	94 ± 88	0.51
HCV RNA (IU/mL) ^†^	1.8×10^6^±1.5×10^6^	1.6×10^6^±1.2×10^6^	2.1×10^6^±1.4×10^6^	0.49
F3-4 Fibrosis stage, n (%)^‡^	11/63 (17.5)	7/51 (13.7)	4/12 (33.3)	0.19
Genotype others, n (%)	9 (1.9)	6 (2.1)	3 (1.5)	
**Propensity score matched patients**	**Total**	**PEG-IFN alfa-2a**	**PEG-IFN alfa- 2b**	P*
Genotype 1, n (%)	248	124	124	0.45
Age, years^†^	50 ± 11	51 ± 11	50 ± 11	0.43
Gender (male), n (%)	148 (59.7)	112 (65.5)	74 (64.3)	0.90
BMI (Kg/m^2^) ^†^	24.4 ± 3.2	24.7 ± 3.2	24.3 ± 3.3	0.38
ALT (IU/L) ^†^	93 ± 76	95 ± 74	92 ± 79	0.83
HCV RNA (IU/mL) ^†^	3.0×10^6^±1.3×10^6^	2.5×10^6^±1.3×10^6^	3.6×10^6^±1.1×10^6^	0.31
F3-4 Fibrosis stage, n (%) ^#^	22/73 (30.1)	18/50 (36.0)	4/23 (18.2)	0.17
Genotype 2/3, n (%)	150	75	75	
Age, years^†^	50 ± 11	48 ± 12	50 ± 11	0.16
Gender (male), n (%)	81 (54.0)	162 (61.8)	108 (62.1)	0.96
BMI (Kg/m^2^) ^†^	23.9 ± 2.9	24.3 ± 3.1	23.6 ± 2.4	0.16
ALT (IU/L) ^†^	89 ± 76	101 ± 88	94 ± 88	0.51
HCV RNA (IU/mL) ^†^	1.7×10^6^±1.4×10^6^	1.6×10^6^±1.2×10^6^	2.1×10^6^±1.4×10^6^	0.49
F3-4 Fibrosis stage, n (%)^¥^	10/46 (21.7)	6/34 (17.6)	4/12 (33.3)	0.42

Abbreviation: *HCV*, hepatitis C virus; *BMI*, body mass index; *ALT*, alanine aminotransferase; PEG-IFN, peginterferon.

*P-value for difference between the PEG-IFN alfa-2a and PEG-IFN alfa-2b groups.

^†^mean ± standard deviation.

^§, ‡ ^Data were available only in 94 and 63 patients, respectively.

^#,¥^Data were available only in 73 and 46 patients, respectively. If the number of patients was smaller than 5, Fisher’s exact test was used.

**Figure 3:**
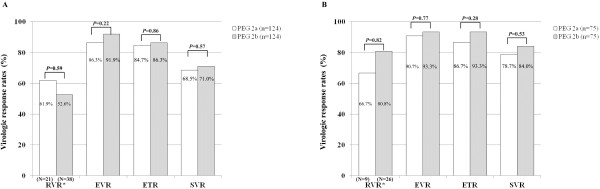
**Analysis in patients with propensity score matching. **RVR, EVR, ETR, and SVR rates were not statistically different between the two PEG-IFN groups in patients with genotype 1 (**A**) and genotype 2/3 (**B**).

To control the effects of potential compounding factors, propensity-score matched analysis for age, gender, HCV genotype, HCV RNA titer, and serum ALT level between two group was performed on 248 patients with genotype 1 (124 patients from each group) and 150 patients with genotype 2/3 (75 patients from each group) (Table 3). Baseline mean age, gender, BMI, serum ALT, and HCV RNA titer were not different between two groups (all *P*-values >0.05) (Table 3). RVR data was available for only 59 (23.8%) patients with genotype 1 and 35 (23.3%) patient with genotype 2/3. RVR, EVR, ETR, and SVR rates were not statistically different between two groups (*P*-values for each >0.05) for HCV genotype 1 (Figure 3A) and genotype 2/3 (Figure 3B), respectively.

### Adverse events

AEs and early drug discontinuation rates are suggested in Table 4. Of the 661 patients, 542 (82.0%) patients experienced at least one treatment-related AE. The Table 4 summarizes the most severe graded AE that individual patients experienced during treatment. Of the 661 patients, 493 (74.6%) experienced a grade 1 or 2 AE and 49 (7.4%) experienced a grade 3 AE without showing a significant intergroup difference (*P*=0.23) (Table 4). The rates of common AEs in the two groups, that is gastrointestinal symptoms, dermatologic symptoms, and emotional friability were not significantly different (*P*-values for each >0.05) and neither were severe hematologic events. Although the rates of flu-like symptoms or alopecia showed statistical difference between two PEG-IFN groups (*P*<0.01), the number of patients with grade 3 was small in each group. Two patients in the PEG-IFN alfa-2a group were died due to infectious colitis or sepsis induced by severe neutropenia.

**Table 4:** Adverse events in all patients with chronic HCV infection

**Variables**	**Total**	**PEG-IFN alfa-2a**	**PEG-IFN alfa-2b**	P*
	**(N=661)**	**(N=402)**	**(N=259)**	
Overall AE, N (%)	542 (82.0)	312 (77.6)	230 (88.8)	
Grade 1-2/3^†^	493/49 (91.0/9.0)	288/24 (92.3/7.7)	205/25 (89.1/10.9)	0.23
Common AE, N (%)				
Flu-like symptoms				
Grade 1-2/3	421/0 (63.7/0)	229/0 (57.0/0)	192/0 (74.1/0)	<0.01
GI symptoms				
Grade 1-2/3	152/11 (23.0/1.7)	82/6 (20.4/1.5)	70/5 (27.0/1.9)	0.12
Dermatologic reactions				
Grade 1-2/3	74/2 (11.2/0.3)	38/1 (9.5/0.2)	36/1 (13.9/0.4)	0.19
Emotional friability				
Grade 1-2/3	183/23 (27.7/3.5)	102/11 (25.4/2.7)	81/12 (31.3/4.6)	0.08
Alopecia				
Grade 1-2/3	96/20 (14.5/3.0)	50/7 (12.4/1.7)	46/13 (17.8/5.0)	<0.01
Hematologic events, N (%)				
ANC, <500/mm^3^	3 (0.5)	0 (0)	3 (1.2)	0.06
Hemoglobin, <8.5 g/dL	25 (3.8)	15 (3.7)	10 (3.9)	1.00
Platelet, <25,000/mm^3^	6 (0.9)	6 (1.5)	0 (0)	0.09
Serious AE, N (%)				
Severe infection or death	2 (0.3)	2 (0.5)	0 (0)	0.52
Patients who did not meet 80/80/80 rule	181 (27.4)	118 (29.4)	63 (24.3)	0.18
Dose modification, N (%)	82 (45.3)	58 (49.23)	24 (38.1)	0.16
Discontinuation, N (%)	99 (54.7)	60 (60.6)	39 (39.4)	0.52
AE/hematologic event	47 (47.5)	27 (45.0)	20 (51.3)	0.68
Non-virologic response	18 (18.2)	10 (16.7)	8 (20.5)	0.79
Incidental severe infection	2 (2.0)	2 (3.3)	0 (0)	0.52
Follow-up loss	32 (32.3)	21 (35.0)	11 (28.2)	0.48

Abbreviation: *HCV*, hepatitis C virus; *AE*, adverse event; *GI*, gastrointestinal; *ANC*, absolute neutrophil count; *PEG-IFN*, peg-interferon; *NA*, not available.

*P-value stands for comparison of the frequency of adverse event or each variable between the PEG-IFN alfa-2a and PEG-IFN alfa-2b groups.

^† ^Most severe grade among adverse events that patients had experienced during treatment.

If the number of patients was smaller than 5, Fisher’s exact test was used.

Of the 661 patients, 181 (27.4%) were non-adherence to treatment, and of these, 82 (45.3%) and 99 (54.7%) experienced dose modification and drug discontinuation, respectively, with no significant intergroup difference (Table 4). The most common cause of early drug discontinuation was a severe AE or hematologic event (47.5%), followed by no virologic response (18.2%) or follow-up loss (32.3%). Causes of early drug discontinuation were similar between two groups.

## Discussion

We found that the efficacy and safety of PEG-IFN alfa-2a in treatment-naïve Korean patients with chronic HCV are not different with those of PEG-IFN alfa-2b, regardless of HCV genotypes, unlike the results of Caucasian studies [13-15]. These findings were confirmed by additional analysis of the patients who sufficiently complied with the treatment schedule (the 80/80/80 rule). Furthermore, propensity score matched analysis showed that SVR rates were similar between two PEG-IFN treatment groups. To the best of our knowledge, this is the largest multicenter study to compare the efficacy and safety between two types of PEG-IFNs in treatment-naïve chronic HCV patients in Asian area where favorable *IL-28B* gene polymorphism is dominant. Although several previous studies have compared the efficacy or safety of these two PEG-IFNs, most of them included a relatively small number of patients and/or were limited to HCV genotype 1 patients, and were single center studies without adjusting for confounders and with including retreated patients [19-22].

Recently, several Western studies reported that the SVR rate of PEG-IFN alfa-2a plus ribavirin is higher than that of PEG-IFN alfa-2b plus ribavirin [13-15]. However, there have been discrepancies between the results of the previous clinical trials of two PEG-IFNs [11-14]. Furthermore, before the present study, there has been no large-scaled multicenter study for comparison of two kinds of PEG-IFNs in terms of SVR rates in treatment-naïve HCV patients in Asian area. Although recent Japanese study showed higher SVR rate in PEG-IFN alfa-2a treatment group as compared to PEG-IFN alfa-2b group, this single center study has limitations of being limited to HCV genotype 1 patients with HCV RNA >5 log IU/mL [19]. Most of all, retreated patients were included, and the SVR rates between two PEG-IFN groups were not different in subgroup analysis according to hepatic fibrosis [19]. Therefore, we think that this result may not be representative of treatment-naive Asian patients with chronic hepatitis C.

In the present study, the overall SVR rates between two PEG-IFNs were not statistically different regardless of HCV genotype. The different outcomes between Western and Korean patients with respect to the efficacy of these two PEG-IFNs can be explained as follows. First, host genetic diversity among different races may have affected the different outcomes because the frequency of the favorable *IL28B* gene polymorphism is higher in Asian patients than in Western patients [23,24]. In fact, it was recently reported that about 95% of Korean HCV patients have favorable *IL28B* genotype (rs12979860 CC) to virologic response [25]. The previous study reported that favorable *IL28B* genotype can be predictors of RVR, which is the strong predictor of SVR in HCV patients [24]. In addition, our subgroup analysis showed that SVR rates were higher in patients who achieved RVR, regardless of HCV genotype. This result indirectly reflects that most of our study subjects enrolled has favorable *IL28B* genotype although we did not explore the *IL28B* genotype due to the absence of stored frozen serum sample of the patients enrolled. Furthermore, the overall RVR and SVR rates achieved by PEG-IFN alfa-2a or alf-2b in the present study were higher than those observed in Western studies [13-15]. This finding suggests that *IL28B* gene polymorphism in Korean patients may be responsible for the similar therapeutic responses between two PEG-IFNs compared to Western data. Second, in previous studies, more than 50% of patients were non-adherent or approximately half of patients required dose reduction of ribavirin [13,14]. Third, the percentage of patients with a body weight of over 75 kg in the present study was lower than that in one Italian study (12-21% *vs.* 33%) [13], and mean patient weight was lower than in the other Italian study (64-67 kg *vs.* 69-72 kg) [14]. Fourth, the present study was investigator-initiated, driven, and concluded.

In the present study, the comparison of RVR rate between two treatment groups could be evaluated only in 20-23% of the enrolled patients because the concept of RVR was devised relatively recently [26]. Interestingly, the RVR rate found in the present study was not significantly different between the two PEG-IFNs groups, regardless of HCV genotype. In addition, we found no significant difference between two PEG-IFN groups in terms of EVR and ETR, regardless of HCV genotype. Therefore, our data suggest that RVR, EVR, and ETR as well as SVR may not be different between two PEG-IFNs in Korean patients with HCV. Moreover, given that the backbone of the anti-HCV therapy in genotype 1 HCV patients is still PEG-IFN despite the recent introduction of direct-acting antiviral agents in these patients [27,28], clinical implication of our data may be important in HCV patients.

Many factors such as age, HCV genotype, viral load, degree of fibrosis [29], and accumulated PEG-IFN plus ribavirin dose [30-33] are known to affect antiviral response to PEG-IFN plus ribavirin. In the present study, baseline characteristics including the proportion of young aged (≤50 years) patients, which is a known good predictor for SVR, were similar between two PEG-IFN groups. Interestingly, the SVR rates of subgroup comparisons based on dichotomizations of age, HCV viral load, and hepatic fibrosis also showed no difference both in patients with HCV genotype 1 and genotype 2/3, respectively (Table 2). However, the number of histologic data in the PEG-IFN alfa-2b group was incidentally smaller than PEG-IFN alfa-2a group despite the statistically insignificance between two PEG-IFN groups. Moreover, the current study was not a randomized trial, and thus, it may have been affected by confounding factors. Therefore, to reduce the effect of possible confounding factors, and we applied propensity-score matching to the two groups. This use of propensity score matching to eliminate confounding factors is a unique strength of the present study as it was not performed in previous retrospective studies [20-22].

Given the different pharmacokinetics and pharmacodynamics of PEG-IFN alfa-2a and alfa-2b [9,10], there could be a possibility that they differ with respect to drug-related AEs. Silva et al. [9] reported that neutropenia and treatment discontinuation due to an AE were more frequent in the PEG-IFN α-2a group. However, Rumi et al. [14] reported similar AE frequencies for the two PEG-IFNs. Similarly, we found no statistical difference between two PEG-IFN groups although about 80% of the enrolled patients experienced a mild to severe AE. We cannot explain the exact cause of statistical indifference between two PEG-IFNs groups except flu-like symptoms or alopecia, but we cautiously supposed that the sample size for the comparative analysis of AE both in the present study and previous studies may be insufficient to detect difference between two groups. Therefore, we suggest that further large-scale studies are warranted to determine this relationship. Moreover, weight-based treatment of PEG-IFN alfa-2b and/or ribavirin may affect these outcomes. Considering AEs induced by high dose of PEG-IFN or ribavirin, weight-based treatment regimen might reduce the incidence of AE and give better tolerance to patients with low body weight than fixed dose regimen.

Our study has several limitations. First, inherent selection bias was present due to the retrospective study design. However, a large number of treatment-naïve patients were analyzed, and propensity score matching was used in order to avoid potential confounding factors. Moreover, most HCV patients in our country have favorable *IL-28B* genotype, and therefore, our results can be applied to treatment-naïve HCV patients in area where the frequency of favorable *IL-28B* genotype is high. Second, dose modification of PEG-IFN or ribavirin and monitoring system for AE were not strictly controlled among centers, but treatment schedules were based on the current guideline [5]. Third, RVR rates were not evaluated for the whole cohort because RVR is a recently introduced concept.

## Conclusion

In conclusions, EVR, ETR, SVR, and the safety of PEG-IFN α-2a were not different with those of PEG-IFN α-2b in treatment-naive Korean patients with chronic HCV, regardless of HCV genotype, unlike the Western data. Our data suggest that either of these two PEG-IFNs can be used in chronically HCV-infected Korean patients, who achieve better outcomes on antiviral treatment than Caucasian patients. We hope that our results will be used to establish a certain guidelines for the treatment of Korean patients infected with chronic hepatitis C.

## Abbreviations

HCV: Hepatitis C virus; PEG-IFN: Pegylated interferon; SVR: Sustained virological response; AASLD: American Association for the Study of Liver Diseases; ALT: Alanine aminotransferase; RVR: Rapid virological response; EVR: Early virological response; ETR: End-of-treatment response; AE: Adverse event; ITT: Intention to treat.

## Competing interests

This study was supported from The Korean Association for the Study of the Liver (KASL) in 2009. The authors have no conflict to declare.

## Authors’ contribution

Y-JJ and J-WL: Conception and design, collection and assembly of data, analysis and interpretation of the data, drafting of the article, provision of study materials or patients, administrative and technical or logistic support. J-IL, SHP, CKP, YSK, S-HJ, YSK, JHK, SGH, KSR, HJY, JYC, SWC, JSL, YMP, JWJ, CKL, JHS, JMY: Collection and assembly of data and provision of study materials or patients. SH: Analysis and interpretation of the data. All authors read and approved the final manuscript.

## Pre-publication history

The pre-publication history for this paper can be accessed here:

http://www.biomedcentral.com/1471-230X/13/74/prepub
